# Differences in objectively measured physical activity and sedentary behaviour between white Europeans and south Asians recruited from primary care: cross-sectional analysis of the PROPELS trial

**DOI:** 10.1186/s12889-018-6341-5

**Published:** 2019-01-21

**Authors:** Gregory J. H. Biddle, Charlotte L. Edwardson, Alex V. Rowlands, Melanie J. Davies, Danielle H. Bodicoat, Wendy Hardeman, Helen Eborall, Stephen Sutton, Simon Griffin, Kamlesh Khunti, Thomas Yates

**Affiliations:** 1 Diabetes Research Centre, University of Leicester, Leicester General Hospital, Leicester, UK; 2 NIHR Leicester Biomedical Research Centre, University of Leicester and University Hospitals of Leicester NHS Trust, Leicester, UK; 3 Behavioural Science Group, Institute of Public Health, University of Cambridge, Cambridge, CB2 0SR UK; 4 School of Health Sciences, University of East Anglia, Research Park, Norwich, NR4 7TJ UK; 5 Department of Health Sciences, University of Leicester, Leicester, UK; 6 Epidemiology Unit, University of Cambridge School of Clinical Medicine, Cambridge, UK; 7 Primary Care Unit, University of Cambridge School of Clinical Medicine, Institute of Public Health, University of Cambridge, Cambridge, UK; 8 NIHR Collaboration for Leadership in Applied Health Research and Care East Midlands, University of Leicester and University Hospitals of Leicester NHS Trust, Leicester, UK

**Keywords:** Sedentary lifestyle, Exercise, Ethnic groups, Primary health care

## Abstract

**Background:**

Self-reported data have consistently shown South Asians (SAs) to be less physically active than White Europeans (WEs) in developed countries, however objective data is lacking. Differences in sedentary time have not been elucidated in this population. This study aimed to quantify differences in objectively measured physical activity and sedentary behaviour between WEs and SAs recruited from primary care and to investigate differences in demographic and lifestyle correlates of these behaviours.

**Methodology:**

Baseline data were utilised from a randomised control trial recruiting individuals identified at high risk of type 2 diabetes from primary care. Light intensity physical activity, moderate-to-vigorous intensity physical activity (MVPA) and steps were measured using the Actigraph GT3X+, while sitting, standing and stepping time were measured using the activPAL3™. Devices were worn concurrently for seven days. Demographic (employment, sex, age, education, postcode) and behavioural (fruit and vegetable consumption, alcohol consumption, smoking status) characteristics were measured via self and interview administered questionnaires.

**Results:**

A total of 963 WE (age = 62 ± 8, female 51%) and 289 SA (age = 55 ± 11, female 43%) were included. Compared to WEs, SAs did less MVPA (24 vs 33 min/day, *p* = 0.001) and fewer steps (6404 vs 7405 per day, *p* ≤ 0.001), but sat less (516 vs 552 min/day, *p* ≤ 0.001) and stood more (328 vs 283 min/day, *p* ≤ 0.001). Ethnicity also modified the extent to which demographic and behavioural factors act as correlates of physical activity and sedentary behaviour. Differences between sex in levels of MVPA and sitting time were greater in SAs compared to WEs, with SA women undertaking the least amount of MVPA (19 min/day), the least sitting time (475 min/day) and most standing time (377 min/day) than any other group. Smoking and alcohol status also acted as stronger correlates of sitting time in SAs compared to WEs. In contrast, education level acted as a stronger correlate of physical activity in WEs compared to SAs.

**Conclusion:**

SAs were less active yet less sedentary than WEs, which demonstrates the need to tailor the behavioural targets of interventions in multi-ethnic communities. Common correlates of physical activity and sedentary behaviour also differed between ethnicities.

**Trial registration:**

ISRCTN83465245 Trial registration date: 14/06/2012.

**Electronic supplementary material:**

The online version of this article (10.1186/s12889-018-6341-5) contains supplementary material, which is available to authorized users.

## Background

The risk of developing chronic diseases such as type 2 diabetes and cardiovascular disease is increased in South Asian (SA) populations relative to a White European (WE) population [1, 2]. Physical activity is a cornerstone of current diabetes prevention and treatment guidelines in the United Kingdom (UK) [3, 4], and differences in physical activity and other health behaviours, such as smoking, between ethnic groups have been suggested as one of the reasons for the disparity in chronic disease risk. For example, SA adults and adolescents self-report lower levels of physical activity than those from a WE background [5–8]. However, assessing differences between groups using self-reported physical activity levels has many limitations. For example, the vast majority of physical activity questionnaires have only been validated in White populations [9], despite the fact that validity is likely to vary depending on the population sampled [10]. It is likely that the biases inherent with self-reported measures differ according to cultural norms and expectations, for instance, it has been suggested that physical activity may be considered unhealthy and may aggravate illnesses further in SA communities [11–13]. Substantial differences were shown in walking and moderate-to-vigorous intensity physical activity (MVPA) by self-report, yet only minimal differences were observed objectively [9]. This highlights the importance of employing objective measurement when assessing differences in physical activity between populations.

Ethnic differences in physical behaviours beyond MVPA have not been well researched, including time spent sedentary, defined as behaviour at low energy expenditure (≤ 1.5 Metabolic Equivalents) in a sitting, lying or reclining posture [14]. Sedentary behaviour is widely considered an independent behaviour to physical activity. Time spent sedentary is associated with increased risk of mortality [15–17], and increased risk of morbidity such as type 2 diabetes and cardiovascular disease [16, 18], independent of physical activity, it therefore may have important implications for minority ethnic health. In the only study comparing sedentary time between ethnic groups to date, differences in objectively measured sedentary time were observed between White Americans, Mexican Americans and Black Americans, with Mexican Americans being the least sedentary group [19]. Further research is needed for other ethnic groups and within other countries.

Previous physical activity research in WEs and SAs has been focused on overall differences in behaviour. Data are also needed on whether the correlates of physical activity and sedentary behaviour differ by ethnic group. Greater understanding of possible correlates of health behaviour is an important step in informing more effective intervention design [20]. Extending the knowledge of key correlates of physical activity and sedentary behaviour to outline any ethnic variations is therefore important to improve the effectiveness of future interventions, specifically in ethnically diverse communities.

Often ethnic differences in health behaviour have been limited to the general population, rather than high risk primary care populations that are most likely to receive and benefit from behaviour change interventions. In particular, diabetes prevention programmes targeting high risk individuals have been introduced in many countries globally and provide a dedicated opportunity for promoting physical activity to large numbers of adults [21, 22]. The largest national prevention programme was recently rolled-out in England with the stated aim of targeting high risk groups and reducing health inequality [22]. A focus on SA populations is particularly important as they are the largest minority ethnic group in the UK, with Indians making up 2.5% of the population and Pakistanis 2.0% [23]. Therefore understanding ethnic differences in the levels and correlates of physical activity and sedentary behaviour, particularly in high risk primary care populations eligible for a diabetes prevention programme, will further help increase the knowledge needed to effectively tailor behavioural prevention programmes to minority groups.

The primary aim of this study was to compare the levels of objectively measure physical activity and sedentary behaviour between WEs and SAs from baseline data of a randomised control trial [24]. The secondary aim was to investigate the extent to which common demographic and behavioural factors act as correlates of physical activity and sedentary behaviour and whether these differ by ethnicity.

## Methods

### Participants

This analysis reports baseline data from the PRomotion Of Physical activity through structured Education with differing Levels of ongoing Support for people at high risk of type 2 diabetes (PROPELS) trial. The PROPELS trial is a multi-centre (Leicester and Cambridge) randomised control trial aimed at increasing physical activity in those at high risk of type 2 diabetes. The PROPLES trial is a four year intervention designed to increase ambulatory activity through structure education, tailored text messages and phone calls. The detailed methods of this study have been reported elsewhere [24]. People were identified from primary care as having glycated haemoglobin (HbA1c test) in the high risk range (≥6.0 to < 6.5%; ≥42 to < 48 mmol/mol) within the past five years [25]. Participants aged 40 to 74 years for WE, aged 25 to 74 years for SA and had access to a mobile phone (and willing to use it for the study) were eligible. The age range differed between WE and SA participants in accordance with National Institute for Health and Care Excellence guidance for the prevention of type 2 diabetes [25], as it is recommended that peopled aged 25–39 of South Asian or any other minority ethnic group should be given a risk assessment for type 2 diabetes. Participants were excluded if they were found to have an HbA1c ≥6.5% (≥48 mmol/mol), were pregnant, unable to take part in ambulatory activity, involved in other related intervention studies, unable to understand basic written and verbal English or unable to give informed consent. The study oversample SAs aiming to make up 20% of the study sample. Ethics approval was granted by the National Health Service (NHS) National Research Ethics Committee, Leicester (04/05/2012, ref.: 12/EM/0151). Participants provided written informed consent.

### Objectively measured physical activity and sedentary behaviour data

Participants were asked to wear two accelerometers (Actigraph GT3X+ and activPAL3™) simultaneously for seven consecutive days. For this study, Actigraph data was used to assess physical activity (i.e. steps, light intensity physical activity and MVPA) and the activPAL device was used for postural outcomes (i.e. sitting, standing and stepping).

The Actigraph GT3X+ (Pensacola, Florida, USA) was worn on the right anterior axillary line above the hip on an elastic belt for seven waking days. Data were collected at a frequency of 100 Hz and reintegrated into 60 s epochs for this analysis using the manufacturer’s software normal filter. At least three valid wear days were required to be included in the analysis. A valid day consisted of at least 600 min of wear time, with non-wear time being defined as a minimum of 60 min of continuous zero counts [26]. Freedson cut-points, applied to the vertical axis (x axis), were used to categorise light intensity physical activity (LPA) (100–1951 cpm) and MVPA (≥1952 cpm) [27]. The cut off for spurious epoch values was ≥30,000. Files were processed using KineSoft V3.3.76; a commercially available analytical software (KineSoft, Loughborough, UK). Output variables included wear time, LPA, MVPA and steps. The ActiGraph GT3X+ has been shown to be a valid and reliable measure for free living physical activity in adult populations [28].

The activPAL3™ (PAL Technologies, Glasgow, UK) was worn on the midline anterior aspect of the upper thigh secured with a hypoallergenic waterproof dressing (Hypafix Transparent). The device was waterproofed by a nitrile sleeve and wrapped in a waterproof dressing (Hypafix Transparent). Participants were asked to wear the device continually for 24 h/day for the same seven days as the Actigraph GT3X+. activPAL data were downloaded using the manufacturer’s software (activPAL Professional Research Edition, PAL Technologies, Glasgow, UK) and processed using a validated automated algorithm in STATA (StataCorp LP, Texas, USA) described in detail elsewhere [29]. In brief, the algorithm uses the activPAL event files to isolate waking hours from ‘sleeping’ (time in bed), prolonged non-wear periods and invalid data. A valid day was defined as a day with < 95% of time spent in any one behaviour (e.g., standing or sitting), > 500 steps and ≥ 10 h of waking hours data [29]. Participants were required to have at least three valid days of data to be included in the analysis. Output variables included waking wear time and time spent in the postures of sitting, standing and stepping. The activPAL is used extensively in sedentary behaviour research and has been shown to be reliable and valid for use in sedentary behaviour measurement [30].

### Demographic and Behavioural data

During baseline visits basic demographic and behavioural information were collected. Data collected were used to define ethnicity (WE and SA). Participants were defined as WE if they reported to be White British, White Irish or any other white background, while SAs was defined when reporting to be Indian, Pakistani, Bangladeshi or any other Asian background. Other demographic data collected were age which was categorised for the purposes of this study (< 65 or ≥ 65 years of age) [31], sex (male or female), self-reported occupation type (predominantly seated, standing, manual or retired/other) and education level (none, GCSE, A-level/college or University). Social deprivation was calculated by assigning an Index of Multiple Deprivation (IMD) score to participant’s home postcodes. Behavioural characteristics collected via self-report (explained in detail previously [24]) were smoking status (current/ex-smoker and never smoked), alcohol consumption (low: drink ≤1 drinks/day on 0–2 days per week; medium: drink 3–4 drinks on 1 day per week or 1–2 drinks on 2–4 days per week; and high: drink on ≥5 days or ≥ 3 drinks on ≥2 days) and fruit and vegetable consumption (low: ≤4 times per week; medium: 5–7 times per week; and high: ≥8 times per week). These data were collected via self-administered and interview-administered questionnaires.

### Statistical analyses

Demographic and behavioural variables are presented as numbers and percentages for each group. Descriptive statistics were calculated for the physical activity and sedentary behaviour variables. All physical activity and sedentary behaviour variables are reported as minutes per day, excluding steps (steps per day). Data are reported as means or marginal means (with 95% confidence intervals). Between groups testing was conducted to compare differences between WEs and SAs in the demographic and behavioural categories. Independent samples t-tests and chi-squared tests were used for continuous and categorical variables respectively.

#### Ethnic differences in physical activity and sedentary behaviour

Analysis of covariance (ANCOVA) analyses were used to quantify the differences in physical activity and sedentary behaviour between ethnicities, whilst adjusting for potential confounders. Two models of adjustment were used. Model 1 adjusted for wear time (Actigraph) or waking wear time (activPAL), number of valid wear days and season of data collection. Model 2 additionally adjusted for age, sex, occupation type, and education level, smoking status and IMD score.

#### Correlates of physical activity and sedentary behaviour

To investigate the extent to which categories of age, sex, employment, education, smoking, alcohol consumption, and fruit and vegetable intake acted as correlates of physical activity and sedentary behaviour, ANCOVA was used. Analyses were adjusted for wear time (Actigraph) or waking wear time (activPAL), number of valid wear days, season of data collection, age, sex, occupation type and education level, unless grouped by said variable. Interaction analyses were conducted to assess whether ethnicity modified these associations. Significant ethnicity interactions were further investigated through stratified analysis. All analysis was 2-sided; *p* < 0.05 was considered significant for main effects and interactions. All statistical analysis was conducted using IBM SPSS Statistics 24.

## Results

### Participants

**Fig. 1:**
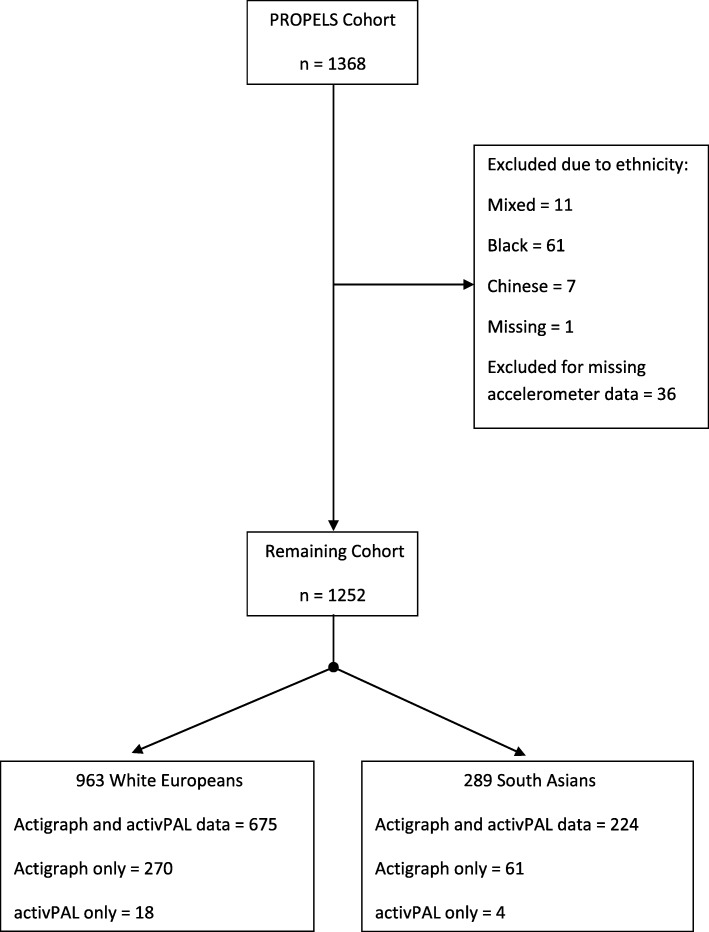
Flow diagram of included participants

Out of the 1368 participants recruited for the study, 1252 were included in the analysis (963 WE; 289 SA). Figure 1 reports the flow of participants and included data. There were no differences in sex, age group and education level between those with missing data and those with complete data. However, WE were more likely to have missing data than SAs (29.9% vs. 22.5%, *p* = 0.014). Missing data are outlined in Additional file 1: Table S1. Table 1 shows the characteristics of included participants, as a whole cohort and stratified by ethnicity. Overall, WEs were older (mean ± SD: 62 ± 8 vs 55 ± 10 years of age), more likely to be female (51% vs 43%), eat high levels of fruit and vegetables (27% vs 19%), consume high levels of alcohol (29% vs 12%), more likely to live in the least deprived area by IMD quintile (30% vs 7%) and be a current or ex-smoker (55% vs 26%) compared to SAs. In addition, SAs were more likely than WEs to engage in standing based occupations (26% vs 15%). The number of participants with valid data from the ActiGraph was greater than the number of participants with valid data from the activPAL.

**Table 1:** Characteristics and descriptive statistics of included participants

Variable	Overall (*n* = 1252)	White European (*n* = 963)	South Asian (*n* = 289)
Age	60 (27–74)	**62 (40–74)**	**55 (27–74)**
Adults (18–64)	826 (66)	**587 (61)**	**239 (83)**
Older Adults (≥65)	426 (34)	**376 (39)**	**50 (17)**
Sex			
Male	640 (51)	**474 (49)**	**166 (57)**
Female	612 (49)	**489 (51)**	**123 (43)**
Occupation			
Sedentary	331 (26)	**262 (27)**	**69 (24)**
Standing	215 (17)	**141 (15)**	**74 (26)**
Manual	156 (13)	**124 (13)**	**32 (11)**
Retired/Other	550 (44)	**436 (45)**	**114 (39)**
Education			
None	263 (22)	209 (22)	54 (19)
GCSE/O Level/GNVQ	296 (24)	226 (24)	70 (25)
A Level/College/City & Guilds	348 (29)	272 (29)	76 (27)
University Degree	315 (26)	234 (25)	81 (29)
IMD Quintiles			
1 (Least deprived)	307 (25)	**288 (30)**	**19 (7)**
2	241 (19)	**204 (21)**	**37 (13)**
3	279 (22)	**202 (21)**	**77 (27)**
4	244 (20)	**146 (15)**	**98 (34)**
5 (Most deprived)	181 (15)	**123 (13)**	**58 (20)**
Fruit and Vegetable Consumption			
Low	108 (9)	**71 (7)**	**37 (13)**
Medium	828 (66)	**632 (66)**	**196 (68)**
High	316 (25)	**260 (27)**	**56 (19)**
Alcohol Consumption			
Low	681 (54)	**461 (48)**	**220 (76)**
Medium	257 (21)	**223 (23)**	**34 (12)**
High	314 (25)	**279 (29)**	**35 (12)**
Smoking Status			
Never Smoked	646 (52)	**432 (45)**	**214 (74)**
Current/ex-smoker	606 (48)	**531 (55)**	**75 (26)**
Physical Activity (ActiGraph)			
Valid Wear Days	6.5 (0.8)	6.5 (0.8)	6.6 (0.8)
Wear Time	884 (82)	**880 (79)**	**898 (89)**
LPA	304 (85)	**300 (84)**	**317 (87)**
MVPA	24 (13; 43)	24 (13; 44)	24 (12; 39)
Steps	7179 (3177)	7235 (3243)	6993 (2948)
Sedentary Behaviour (activPAL)			
Valid Wear Days	6.6 (0.7)	**6.5 (0.8)**	**6.7 (0.7)**
Wake Time	948 (67)	**944 (64)**	**959 (74)**
Sitting time	543 (113)	**552 (111)**	**513 (116)**
Standing time	295 (97)	**281 (92)**	**335 (103)**
Stepping time	111 (41)	111 (42)	111 (41)

Data as number (%), age is reported as mean (lowest-highest). Physical activity and sedentary behaviour data as mean (±SD), with the exception of MVPA which was not normally distributed, therefore is presented as median (IQR). Bold values represent a significant difference between White Europeans and South Asians

### Ethnic differences in physical activity and sedentary behaviour

Table 2 shows the marginal means for the physical activity and sedentary behaviour variables stratified by ethnicity, adjusting for wear time (ActiGraph), waking wear time (activPAL), number of valid wear days, season of data collection, age, sex, occupation, education, smoking status and IMD score (Model 2). Within the ActiGraph data, WEs performed more MVPA ([mean difference [95% CI]] 9 min [5; 12], *p* ≤ 0.001) and more steps per day than SAs (1001 steps [543; 1460], p ≤ 0.001). Within the activPAL data, WEs showed greater time spent sitting (36 min [17; 54], p ≤ 0.001), less time spent standing (46 min [30; 61], p ≤ 0.001) and spent more time stepping (11 min [5; 18], *p* = 0.001) than SAs.

**Table 2:** Differences between ethnic group’s physical activity and sedentary behaviour variables

Variable	n	White European	n	South Asian	*P*-value
Actigraph	*945*		*285*		
LPA (mins)		304(299–309)		304 (295–314)	0.575
MVPA (mins)		33 (31–35)		24 (21–28)	**< 0.001**
Steps		7405 (7201–7610)		6404 (6013–6796)	**< 0.001**
activPAL	*693*		*228*		
Sitting Time (mins)		552 (544–561)		516 (501–532)	**< 0.001**
Standing Time (mins)		283 (276–290)		328 (315–341)	**< 0.001**
Stepping Time (mins)		114 (111–117)		102 (96–108)	**0.001**

Data as a marginal mean (95% confidence interval). Adjusted for wear time (Actigraph), waking wear time (activPAL), number of valid wear days (both devices), season of data collection, age, sex, occupation type, education, smoking status and IMD score. Mean (SD) wear time values for White Europeans and South Asians were 880 (79.4) and 898 (88.7) minutes respectively. Average wake time values for White Europeans and South Asians were 944 (64.3) and 959 (74.1) minutes respectively. *LPA* Light intensity Physical Activity, *MVPA* Moderate to Vigorous intensity Physical Activity

Significant differences (≤0.05) are highlighted by *P*-vlaues in bold

Data without adjustment for demographic factors (Model 1) are shown in Additional file 1: Table S2. Briefly, differences were still observed for steps (7275 [7079, 7471] vs 6860 [6502, 7218], *p* = 0.047), sitting time (553 min [545, 562] vs 509 [495, 524], p ≤ 0.001) and standing time (283 min [276, 290] vs 330 [318, 342], p ≤ 0.001). No differences were observed for LPA, MVPA or stepping time.

### Correlates of physical activity and sedentary behaviour

**Fig. 2:**
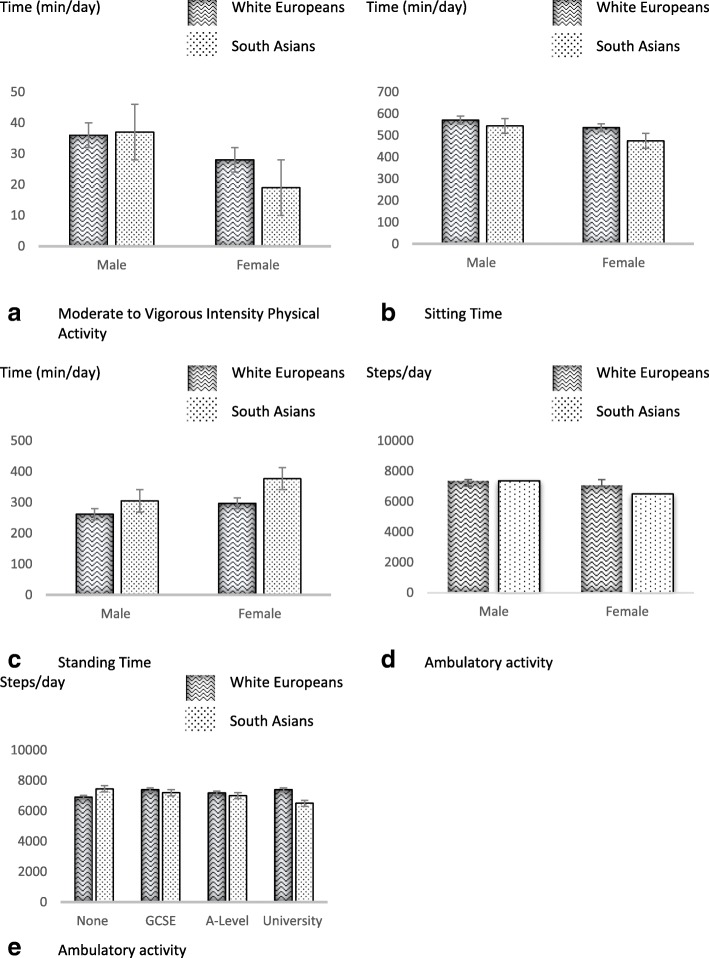
Demographic ethnicity interactions Wavy lines: White Europeans, Spots: South Asians Data displayed as marginal means with error bars displaying standard errors; full data is presented in Additional file 1: Table S3

Table 3 shows the association of different demographic characteristics with physical activity and sedentary behaviour in the combined study cohort. Being older was associated with less LPA, MVPA, stepping time and total steps. Being male was associated with higher MVPA but lower LPA and standing with more sitting. Occupation type and education level showed differing associations with physical activity and sedentary behaviour, with those in sedentary jobs doing the most sitting and least LPA, MVPA, steps and standing, while those with university education had higher sedentary time but also higher LPA. Interaction analysis revealed that ethnicity modified some associations, outlined in Table 3. The direction of the significant interactions is displayed in Fig. 2. Differences between men and women in MVPA, sitting and standing time were greater in SAs than WEs. In contrast, education level was more strongly associated with steps in WEs compared to SAs.

**Table 3:** Demographic differences in physical activity and sedentary behaviour and interactions with ethnicity

	Actigraph			activPAL		
	LPA	MVPA	Steps	Sitting Time	Standing Time	Stepping Time
Occupation						
Sedentary	268 (259; 277)	29 (26; 32)	6352 (5971; 6733)	591 (577: 607)	257 (244; 270)	98 (92; 104)
Standing	319 (308; 329)	31 (27; 35)	7511 (7081; 7942)	502 (485; 518)	326 (313; 340)	119 (113; 125)
Manual	342 (329; 355)	33 (29; 38)	8214 (7680; 8747)	512 (491; 533)	312 (294; 330)	125 (117; 133)
Retired/Other	308 (300; 316)	31 (29; 34)	7200 (6860; 7541)	540 (526; 554)	297 (285; 308)	111 (106; 116)
*p*-value^a^	**≤0.001**	0.190	**≤0.001**	**≤0.001**	**≤0.001**	**≤0.001**
Interaction *p*-value^b^	0.890	0.092	0.159	0.878	0.533	0.680
Sex						
Male	289 (283; 295)	36 (34; 38)	7368 (7124; 7612)	563 (553; 573)	273 (265; 282)	111 (108–115)
Female	319 (313; 325)	26 (24; 28)	6946 (6698; 7193)	523 (513; 533)	314 (306; 323)	110 (106–114)
*p*-value^a^	**≤0.001**	**≤0.001**	**0.020**	**≤0.001**	**≤0.001**	0.645
Interaction *p-*value^b^	0.848	**0.008**	0.037	**0.047**	**0.006**	0.575
Age						
Adults	309 (303; 314)	34 (32–36)	7484 (7259; 7710)	539 (530–548)	295 (288; 303)	114 (110; 117)
Older Adults	294 (286; 302)	26 (23–29)	6562 (6300; 6894)	551 (538–565)	292 (280; 303)	105 (100–110)
*p*-value^a^	**0.007**	**≤0.001**	**≤0.001**	0.158	0.614	**0.010**
Interaction *p*-value^b^	0.676	0.077	0.121	0.514	0.780	0.265
Education						
None	318 (309; 328)	29 (26; 32)	6985 (6595; 7375)	549 (534; 564)	290 (278; 303)	109 (104; 115)
GCSE	309 (300; 318)	31 (28; 34)	7403 (7053; 7752)	532 (518; 546)	301 (290; 313)	116 (110; 121)
A-level/College	307 (299; 315)	31 (29; 34)	7145 (6819; 7471)	537 (523; 550)	300 (288; 311)	112 (107; 117)
University	283 (275; 292)	33 (30; 36)	7132 (6782; 7483)	556 (542; 571)	284 (272; 296)	107 (101; 112)
*p*-value^a^	**≤0.001**	0.080	0.444	0.056	0.137	0.085
Interaction *p*-value^b^	0.591	0.154	**0.048**	0.076	0.126	0.209

Data as a marginal mean (95% confidence interval)

Model 2: ^a^Testing difference between groups, adjusted for wear time (Actigraph), wake time (activPAL), number of valid wear days (both devices), season of data collection, ethnicity, age, sex, occupation type, education, smoking status and IMD score (unless grouped by variable)

^b^Ethnicity interaction, adjusted for wear time (Actigraph), wake time (activPAL), number of valid wear days (both devices), season of data collection, age, sex, occupation type, education, smoking status and IMD score (unless grouped by variable)

*LPA* Light intensity Physical Activity, *MVPA* Moderate to Vigorous intensity Physical Activity

Bold values highlight statistical significance of ≤0.05

**Fig. 3:**
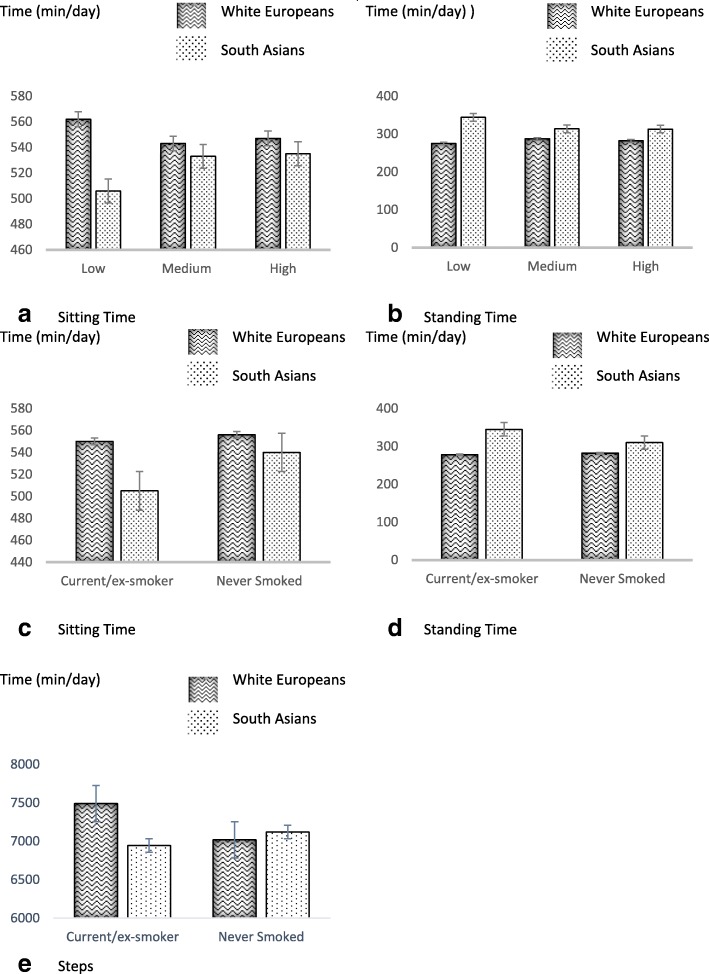
Behavioural ethnicity interactions Low, Medium and High: Alcohol Consumption Data displayed as marginal means with error bars displaying standard errors; full data is presented in Additional file 1: Table S3

Table 4 shows the association of different behavioural characteristics with physical activity and sedentary behaviour. High fruit and vegetable consumption was associated with more MVPA, stepping time and total steps. High alcohol consumption was associated with more MVPA and total steps, while having never smoked was associated with greater stepping time and total steps. Interaction analysis revealed that ethnicity modified some of these associations. Significant interactions are displayed in Fig. 3. Low alcohol consumption and having never smoked were more predictive off less sitting and more standing time in SAs compared to WEs.

**Table 4:** Behavioural differences in physical activity and sedentary behaviour and interactions with ethnicity

	Actigraph			activPAL		
	LPA	MVPA	Steps	Sitting Time	Standing Time	Stepping Time
Fruit and Vegetable Consumption						
Low	304 (293; 315)	26 (22; 30)	6634 (6184; 7083)	549 (531; 567)	294 (279; 309)	105 (99–112)
Medium	300 (294; 307)	31 (28–33)	7083 (6817–7349)	548 (537; 559)	292 (283; 301)	107 (103; 111)
High	307 (301; 314)	34 (31–36)	7455 (7186–7725)	537 (526; 547)	296 (287; 305)	116 (112; 120)
*p*-value^a^	0.332	**0.003**	**0.008**	0.292	0.825	**0.002**
*p*-value^b^	0.401	0.795	0.918	0.326	0.350	0.392
Alcohol Consumption						
Low	301 (295; 307)	29 (27; 31)	6923 (6682; 7163)	547 (537; 556)	294 (286–302)	108 (105; 112)
Medium	307 (298; 317)	31 (27; 34)	7264 (6881; 7647)	536 (521; 552)	298 (385; 311)	114 (108; 120)
High	307 (299; 316)	35 (32; 38)	7641 (7280; 8002)	541 (526; 556)	292 (279; 304)	114 (109; 120)
*p*-value^a^	0.381	**0.015**	**0.007**	0.513	0.798	0.123
*p*-value^b^	0.765	0.945	0.850	**0.006**	**0.002**	0.815
Smoking Status						
Never Smoked	303 (297; 309)	33 (31–35)	7365 (7116; 7615)	537 (527–547)	296 (288; 304)	115 (111–118)
Current/ex-smoker	305 (298–311)	29 (27–31)	6967 (6711; 7222)	550 (540–561)	291 (283; 300)	107 (103–110)
*p*-value^a^	0.767	**0.001**	**0.035**	0.087	0.435	**0.005**
*p*-value^b^	0.444	0.060	**0.050**	**0.037**	**0.002**	0.290

Data as marginal mean (95% confidence interval)

Model 2: ^a^Adjusted for wear time (Actigraph), wake time (activPAL), number of valid wear days (both devices), season of data collection, Ethnicity, Age, Sex, Occupation type and Education (unless grouped by variable)

^b^Ethnicity interaction, adjusted for wear time (Actigraph), wake time (activPAL), number of valid wear days (both devices), season of data collection, Age, Sex, Occupation type and Education (unless grouped by variable)

*LPA* Light intensity Physical Activity, *MVPA* Moderate to Vigorous intensity Physical Activity

Bold values highlight statistical significance of ≤0.05

## Discussion

This paper shows novel differences in objectively measured physical activity and sedentary behaviour between WEs and SAs with a high risk of type 2 diabetes recruited from primary care. WEs did more daily MVPA (+  9 min) and steps (+ 1001), but more sitting (+ 36 min) and less standing (− 46 min) per day compared to SAs, following adjustment for potential confounders (including occupation type). Ethnicity also modified the extent to which common demographic and behavioural characteristics acted as correlates of physical activity; for example, the difference between men and women in levels of habitual MVPA and sitting time were more pronounced in SAs than in WEs, with SA women being the least active but least sedentary group (MVPA = 19 mins/day, sitting time = 475 mins/day), while WE men were the most active and most sedentary (MVPA = 36 mins/day, sitting time = 571 mins/day). To our knowledge, this is the first study to utilise two concurrent well validated and reliable objective measures of both physical activity and sedentary behaviour in an ethnically diverse primary care cohort.

Previous studies have suggested large clinical differences in self-reported physical activity between WEs and SAs, with one study showing that SAs accumulate 35–40% less activity in the form of walking and MVPA [9]. The evidence of differences between WEs and SAs in objectively measure physical activity compared to self-reported data has been more equivocal with some studies reporting differences [32], while others report no differences [9]. The current findings suggest that although there are differences between WEs and SAs in physical activity when measured objectively, the differences are less than in previous self-report studies, although SA women remained the least active group in our cohort. A review of qualitative studies has identified a number of possible explanations as to why SAs are less active, from disliking available structured exercises to prioritising social occasions and modesty based in religious beliefs [33], suggesting that the ethnic differences seen here may result from cultural differences in the way physical activities are conceptualised. Cultural norms may have a particular impact on SA women who are more likely to have cultural expectations for remaining indoors, which acts as a barrier to purposive physical activity [33].

There is a paucity of evidence about differences in sedentary behaviour between ethnic groups, specifically between WEs and SAs. This is important as SAs form the largest minority ethnic group in the UK [34]. Evidence from the USA shows similar differences between ethnic groups, with Whites having higher sedentary time than Mexican-Americans [19]. Evidence to date would therefore suggest that although WEs tend to be the most physically active ethnic group, they are also the most sedentary. In the current study, sitting time was lower in SAs compared to WEs, particularly in women, and correspondingly standing time was greater in SAs compared with WEs. Cultural norms that disincentives physical activity in SA communities may also lead to reduced sedentary time. For example, traditional views of family life with women expected to undertake domestic responsibilities and family care have been noted as the norm in many SA communities and may result in lower levels of sitting time and higher standing time [33, 35, 36]. Different educational levels and employment types may also lead to occupations requiring less sitting time being more common among SAs. However, differences between ethnic groups were maintained in this study after adjustment for educational level and occupational type. More qualitative research and detailed quantitative analyses in relation to time of day and concurrent activities is needed to fully understand the reason for differences in physical activity and sedentary behaviours between ethnicity. Nonetheless, these results do suggest that targets for behavioural interventions may need some degree of tailoring when delivered in multi-ethnic communities. WEs may benefit from interventions that specifically incorporate targets to reduce sedentary time, whereas SAs may benefit more from interventions with a primary focus on increasing physical activity, particularly MVPA. Importantly, these suggestions don’t mean interventions should only focus solely on sedentary behaviour and physical activity for SAs and WEs respectively, but may benefit from a slightly different focus.

The differences reported here between WEs and SAs, particularly in terms of sitting and standing time warrants further investigations to determine the clinical benefit of sitting less and standing more. Current epidemiological and experimental evidence is mixed in relation to standing and its effect on health [37–46]. For example, Henson et al. showed a 34% reduction in glucose incremental area under the curve when sitting was broken up with five minutes of standing every 30 min [40], whereas others (Bailey et al., Pulsford et al) showed no difference in glucose when sitting was broken with standing [37, 43]. However, associations have been consistently reported between sedentary behaviour and increased risk of morbidity and mortality [15, 16, 18, 47, 48], therefore more evidence is needed to identify ways to reduce the increase in risk associated with sedentary behaviour. Although SAs were less sedentary than WEs, greater sedentary time is associated with cardiometabolic diseases and markers of disease among SAs [49], which suggests benefits may still be seen by further reducing sedentary time in SAs, as well as increasing physical activity.

This study also tested for common demographic and behavioural correlates of physical activity, with findings consistent with previous research [20]. However, we extend previous observations by reporting the novel findings that ethnicity modifies the strength of associations of some factors with physical activity and sedentary behaviour. For example, differences between men and women in levels of MVPA and sitting time were greater in SAs compared to WEs. In addition, smoking status and alcohol consumption also acted as stronger correlates of sitting time in SAs compared to WEs. In contrast, education level acted as a stronger correlate of physical activity in WEs compared to SAs. These findings could help identify key groups within each ethnicity that are most likely to benefit from interventions aimed at increasing physical activity or reducing sedentary behaviour. Interestingly, healthy behaviours (i.e. low alcohol consumption and having never smoked) seem to cluster in SAs compared to WEs. This is apparent in Fig. 3 where the least sedentary groups are SAs who have never smoked and who consumer a high level of fruit and vegetables. However, more evidence is needed to identify specific groups and settings where interventions may be most efficient, with particular focus on correlates outlined here within each ethnicity.

This study has a number of strengths and limitations. Strengths include a large sample from primary care and objective measures of physical activity and sedentary behaviour, specifically two different types of accelerometer which were used to accurately capture both domains of physical activity and sedentary behaviour. The high-risk nature of the cohort is both a strength and limitation in that our results may be generalizable to diabetes prevention programmes but not necessarily to the general population. This population may also be more sedentary and less active than the general population. Therefore, these findings should be viewed with caution in relation to a ‘healthy’ population. Self-reported data, such as occupational activity, may have resulted in some residual confounding which may reflect some of the difference in physical activity and sedentary behaviour between WEs and SAs. Other limitations of the study are the disparity in size of the ethnic groups which may affect the power and precision of the effect estimates and that participants were recruited for a clinical trial with a focus on increasing physical activity, which may appeal to those interested in increasing physical activity.

## Conclusions

This study found differences in objectively measured physical activity and sedentary behaviour between WEs and SAs with a high risk of type 2 diabetes, with WEs being the most physically active, while SAs were the least sedentary. This suggests that the relationship between ethnicity and health behaviour is more nuanced than previously suggested, with important consequences for future intervention design and targets. To the authors’ knowledge this is the first study to analyse differences in both objectively measured physical activity and sedentary behaviour between these ethnic groups in a cohort recruited from primary care. Furthermore, the extent to which many common demographic and behavioural factors acted as correlates of physical activity and sedentary behaviour differed by ethnic group. These findings suggest a need to tailor the behavioural targets used in physical activity interventions when designed for and implemented in a multi-ethnic population within primary care, with a physical activity or sedentary behaviour focus for SAs and WEs respectively. Importantly, future research must continue to further understand the relationship between ethnicity and physical activity and sedentary behaviours and the impact this has one health. Illuminating and expanding on these findings with both qualitative research and detailed quantitative analyses to better understand the context in which these behaviours occur, the important influences and the impact these have on health would also be beneficial.

## Additional file

Additional file 1: **Table S1.** Missing data. **Table S2.** Differences between ethnic group’s physical activity and sedentary behaviour variables (marginal means 95% CI.) **Table S3.** Data forming Figs. 2 and 3. (DOCX 19 kb)

## Abbreviations

ANCOVA: Analysis of covariance
CI: Confidence Interval
LPA: Light intensity physical activity
MVPA: Moderate to vigorous physical activity
NHS: National health service
PROPELS: PRomotion of physical activity through structured Education with differing Levels of ongoing Support for people at high risk of type 2 diabetes
SA: South asian
SD: Standard deviation
WE: White european
