# Supplementary material for: Differences in objectively measured physical activity and sedentary behaviour between white Europeans and south Asians recruited from primary care: cross-sectional analysis of the PROPELS trial
Source: BMC Public Health. 2019 Jan 21;19:95. doi: 10.1186/s12889-018-6341-5 (PMC6341710; doi:10.1186/s12889-018-6341-5)
Supplement: Supplementary file 1 — Table S1. Missing data. Table S2. Differences between ethnic group’s physical activity and sedentary behaviour variables (marginal means 95% CI.) Table S3. Data forming Figs. 2 and 3. (DOCX 19 kb) [file 12889_2018_6341_MOESM1_ESM.docx]

**Supplementary Table 1: Missing data**

|  | **Complete Data** | **Missing Data** | **p-value** |
| --- | --- | --- | --- |
| **Ethnicity** |  |  |  |
| White European | 675 (75.1) | 288 (81.6) | 0.283 |
| South Asian | 224 (24.9) | 65 (18.4) |  |
| **Sex** |  |  |  |
| Male | 451 (50.2) | 189 (53.5) | **0.010** |
| Female | 448 (49.8) | 164 (46.5) |  |
| **Age Group** |  |  |  |
| Adults (18-64) | 596 (66.3) | 230 (65.2) | 0.702 |
| Older Adults (≥65) | 303 (33.7) | 123 (34.8) |  |
| **Education** |  |  |  |
| None | 195 (22.2) | 68 (19.8) | 0.391 |
| GCSE/O Level/GNVQ | 214 (24.4) | 82 (23.8) |  |
| A Level/College/City & Guilds | 244 (27.8) | 104 (30.2) |  |
| University Degree | 225 (25.6) | 90 (26.2) |  |
| **Occupation** |  |  |  |
| Sedentary | 242 (26.9) | 89 (25.2) | **0.046** |
| Standing | 169 (18.8) | 46 (13.3) |  |
| Manual | 110 (12.2) | 46 (13.3) |  |
| Retired or Other | 378 (42.0) | 172 (48.7) |  |
| **Fruit and Vegetable Consumption** |  |  |  |
| Low | 80 (8.9) | 28 (7.9) | 0.624 |
| Medium | 190 (21.1) | 234 (66.3) |  |
| High | 208 (23.1) | 91 (25.8) |  |
| **Alcohol Consumption** |  |  |  |
| Low | 501 (55.7) | 180 (51.0) | **0.032** |
| Medium | 190 (21.1) | 67 (19.0) |  |
| High | 208 (23.1) | 106 (30.0) |  |
| **Smoking Status** |  |  |  |
| Never Smoked | 484 (53.8) | 162 (45.9) | **0.011** |
| Current/ex-smoker | 415 (46.2) | 191 (54.1) |  |

Data as number (%). P-value showing difference between groups for missing data.

Supplementary Table 2: Differences between ethnic group’s physical activity and sedentary behaviour variables (marginal means 95% CI)

| **Variable** | **n** | **White European** | **n** | **South Asian** | ***P-value*** |
| --- | --- | --- | --- | --- | --- |
| **Actigraph** | *945* |  | *285* |  |  |
| LPA  (mins) |  | 302  (297-307) |  | 311  (302-320) | 0.084 |
| MVPA  (mins) |  | 32  (30.0-33.3) |  | 29  (25.9-31.9) | 0.959 |
| Steps |  | 7275  (7079-7471) |  | 6860  (6502-7218) | **0.047** |
|  |  |  |  |  |  |
| **activPAL** | *693* |  | *228* |  |  |
| Sitting Time  (mins) |  | 553  (545-562) |  | 509  (495-524) | **<0.001** |
| Standing Time  (mins) |  | 283  (276-290) |  | 330  (318-342) | **<0.001** |
| Stepping Time  (mins) |  | 112  (109-115) |  | 108  (102-113) | 0.214 |
| Model 1: Adjusted for wear time (Actigraph), waking wear time (activPAL), number of valid wear days (both devices) and season of data collection. Average wear time values for White Europeans and South Asians were 880 (79.4) and 898 (88.7) minutes respectively. Average wake time values for White Europeans and South Asians were 944 (64.3) and 959 (74.1) minutes respectively. | | | | | |

| **Supplementary Table 3: Data forming figure 2 and 3**  Model 2: Adjusted for wear time (Actigraph), wake time (activPAL), number of valid wear days (both devices), season of data collection, Age, Sex, Employment status and Education (unless grouped by variable). | | | | | | | | | | | | | | | | | | | | | |
| --- | --- | --- | --- | --- | --- | --- | --- | --- | --- | --- | --- | --- | --- | --- | --- | --- | --- | --- | --- | --- | --- |
|  | **Education** | | | | | | | | | | | | | | | | | | | | |
|  | **White European** | | | | | | | | | | | **South Asian** | | | | | | | | | |
|  | **None** | **GCSE** | | | **A-level** | | **University** | | | ***p-value*** | | **None** | **GCSE** | | | **A-level** | | **University** | | | ***p-value*** |
| **Actigraph** |  |  | | |  | |  | | |  | |  |  | | |  | |  | | |  |
| Steps | 6902 (6454; 7350) | 7401 (6994; 7806) | | | 7180 (6807; 7553) | | 7398 (6986; 7810) | | | 0.354 | | 7453 (6650; 8257) | 7196 (6497; 7895) | | | 7004 (6339; 7669) | | 6505 (5834; 7175) | | | 0.340 |
|  | **Alcohol Consumption** | | | | | | | | | | | | | | | | | | | | |
|  | **White European** | | | | | | | | | | **South Asian** | | | | | | | | | | |
|  | **Low** | | **Medium** | | | **High** | | | ***p-value*** | | **Low** | | | **Medium** | | | **High** | | | ***p-value*** | |
| **activPAL** |  | |  | | |  | | |  | |  | | |  | | |  | | |  | |
| Sitting | 562 (550; 573) | | 543 (527; 560) | | | 547 (531-563) | | | 0.143 | | 507 (491-523) | | | 533 (491; 575) | | | 535 (493; 576) | | | 0.322 | |
| Standing | 275 (266; 285) | | 287 (274; 301) | | | 282 (269; 295) | | | 0.365 | | 344 (331; 358) | | | 314 (278; 351) | | | 313 (277; 348) | | | 0.141 | |
|  | **Gender** | | | | | | | | | | | | | | | | | | | | |
|  | **White European** | | | | | | | | | | **South Asian** | | | | | | | | | | |
|  | **Male** | | | **Female** | | | | ***p-value*** | | | **Male** | | | | **Female** | | | | ***p-value*** | | |
| **Actigraph** |  | | |  | | | |  | | |  | | | |  | | | |  | | |
| MVPA | 36 (33; 38) | | | 28 (25; 30) | | | | **<0.001** | | | 36 (33-41) | | | | 19 (15-24) | | | | **<0.001** | | |
| Steps | 7378 (7089; 7667) | | | 7080 (6799; 7362) | | | | 0.157 | | | 7356 (6888; 7824) | | | | 6492 (5936; 7047) | | | | **0.026** | | |
| **activPAL** |  | | |  | | | |  | | |  | | | |  | | | |  | | |
| Sitting | 571 (559; 583) | | | 536 (525; 548) | | | | **<0.001** | | | 544 (525; 563) | | | | 475 (454; 497) | | | | **<0.001** | | |
| Standing | 262 (252; 271) | | | 297 (288; 306) | | | | **<0.001** | | | 305 (288; 321) | | | | 377 (358; 395) | | | | **<0.001** | | |
|  | **Smoking Status** | | | | | | | | | | | | | | | | | | | | |
|  | **Current/ex-smoker** | | | **Never Smoked** | | | | ***p-value*** | | | **Current/ex-smoker** | | | | **Never Smoked** | | | | ***p-value*** | | |
| **Actigraph** |  | | |  | | | |  | | |  | | | |  | | | |  | | |
| Steps | 7018 (6749; 7287) | | | 7489 (7184; 7794) | | | | **0.026** | | | 7119 (6414; 7825) | | | | 6944 (6539; 7349) | | | | 0.681 | | |
| **activPAL** |  | | |  | | | |  | | |  | | | |  | | | |  | | |
| Sitting | 550 (538; 563) | | | 556 (544; 567) | | | | 0.552 | | | 505 (489; 521) | | | | 541 (511; 570) | | | | **0.047** | | |
| Standing | 278 (268; 288) | | | 282 (272; 291) | | | | 0.589 | | | 345 (331-358) | | | | 310 (285; 336) | | | | **0.024** | | |
